# Shear-Induced Graphitization in Tongyuanpu Shear Zone, Liaodong Peninsula of Eastern China: Insights from Graphite Occurrences, Nanostructures and Carbon Sources

**DOI:** 10.3390/nano15231778

**Published:** 2025-11-26

**Authors:** Mengyan Shi, Nannan Cheng, Jianbin Li, Quanlin Hou, Qianqian Guo, Jienan Pan

**Affiliations:** 1 School of Resources and Environment, Henan Polytechnic University, Jiaozuo 454003, China; shimengyan@hpu.edu.cn (M.S.); lijianbin@home.hpu.edu.cn (J.L.); jn_pan@hpu.edu.cn (J.P.); 2 Henan Key Laboratory of Coal Measure Unconventional Resources Accumulation and Exploitation, Jiaozuo 454003, China; 3 College of Earth and Planetary Sciences, University of Chinese Academy of Sciences, Beijing 100049, China; quhou@ucas.ac.cn (Q.H.); guoqqyj@ucas.ac.cn (Q.G.)

**Keywords:** shear zones, graphitization, nanostructures, carbon isotope, raman thermometry

## Abstract

An in-depth study of the genetic mechanisms of graphite in shear zones is crucial for understanding crustal weakening and the origins of inorganic carbon. This research focuses on mylonitic marble (MM) and cataclastic marble (CM) from the Tongyuanpu shear zone of Eastern China. The occurrences, nanostructures, carbon sources, and genesis of graphite were systematically investigated through micro- to ultra-microscale analysis. The results reveal that the MM contains two graphite varieties: C-foliation-aligned bands and stylolite-derived serrated aggregates. Both exhibit strong *Z*-axis LPO, indicating a deformation temperature below 200 °C. In contrast, the CM features individual graphite particles within fragmented grains. Near-ideal graphite structures are characterized in both types; however, a higher TOC content and a greater graphitization degree are observed in the CM. Raman thermometry indicates metamorphic peak temperatures of 588–673 °C (MM) and 540–682 °C (CM), with the former showing a significant discrepancy from the EBSD results. The δ^13^C_ORG_ values (−12.21‰ to −8.06‰) suggest fluid-derived carbon sources. We propose that reduction reactions involving high-temperature metamorphic fluids supplied the essential carbon source. Ductile shearing accelerated the graphitization of these carbonaceous materials through the accumulation of local strain energy, while subsequent brittle deformation with frictional sliding further facilitated structural transformation.

## 1. Introduction

As localized high-strain zones with significantly higher strain than the surrounding rocks, shear zones are widely developed in various tectonic environments and play a crucial role in crustal weakening and lithospheric rheology [1,2]. The mechanical properties and deformation behavior of rock minerals at different tectonic levels strongly influence the deformation mechanisms and rheological characteristics of shear zones. For example, brittle fracturing and cataclasis dominate in the shallow crust (<10 km); the plastic deformation of mica and quartz is typical in the middle crust (10–20 km); plagioclase governs the plastic deformation in the lower crust (25–35 km); and the plastic deformation of the upper mantle mainly involves olivine (>35 km) [1,3,4].

During detailed fieldwork, it has been observed that graphite is often found in shear zones, especially in high-strain shear zones. For example, graphite frequently occurs in shallow crustal brittle shear zones or faults, particularly those associated with seismic activity [5,6,7]. These graphite are mainly filled in cataclasites or fault gouges in the form of matrix [8,9]. In contrast, in deep crustal ductile shear zones, banded graphite aggregates are primarily hosted within the matrix of mylonitized marbles, schists, or gneisses and exhibit significant lattice preferred orientation [10]. As a unique solid lubricant, graphite possesses an extremely low friction coefficient (~0.1) and can significantly influence the mechanical properties and deformation behavior of shear zones. It is a key factor affecting crustal weakening, seismic activity, and the lithospheric carbon cycle, often referred to as “fossilized fault traces” [9,11,12,13,14,15]. Consequently, the carbon sources, genesis, and the influence of graphite on crustal weakening in shear zones have long attracted extensive attention. Previous studies have revealed that the formation of graphite in shear zones is often associated with the reduction in high-temperature fluids. For example, graphite can form through reactions between high-temperature CO_2_-rich fluids from the deep Earth and reducing gases (such as CH_4_ and H_2_), as exemplified by the reaction CO_2_ + CH_4_ = 2C + 2H_2_O [9,11,16,17]. Other studies suggest that in seismic-related fault activities, instantaneous frictional heat generated by intense shear sliding can lead to the graphitization of low-crystallinity carbonaceous materials, thereby forming graphite [7,18].

Furthermore, field studies indicate that graphite in shear zones predominantly occurs in carbonate rocks, such as limestone or marble. These rocks possess substantial inherent carbon reserves, raising widespread interest in whether they have a genetic link with graphite [8,9,10,19,20]. High-pressure and high-temperature experiments on siderite (1–6 GPa, 600–1200 °C) have shown that it can be reduced to graphite under low-oxygen fugacity conditions, accompanied by the generation of light hydrocarbon gases [21]. However, the experimental temperatures in these studies were significantly higher than the deformation temperatures of natural carbonate shear zones (~200 °C, based on data from references [22,23]), and the influence of shear deformation on graphitization was not considered. In contrast, rotational shear friction experiments conducted at room temperature have demonstrated that shear stress under reducing conditions facilitates the transformation of carbonate minerals into carbonaceous materials [23,24]. Based on these findings, some researchers propose that intense shear stress in shear zones not only induces rock deformation but also triggers tectonic stress chemical reactions that accelerate the graphitization process [20,25,26]. Nevertheless, the detailed process and potential reaction mechanisms remain unclear.

The aforementioned studies demonstrate that considerable debate persists concerning the genesis of graphite in shear zones, which significantly hinders a comprehensive understanding of crustal weakening and in-depth exploration of inorganic carbon origins. In the marble shear zone of Tongyuanpu area, located on the Liaodong Peninsula in Eastern China, graphite occurs in both mylonitic marble (MM) and cataclastic marble (CM). A detailed investigation into the occurrences and genesis of graphite within these two tectonites—characterized by distinct deformation mechanisms—offers critical insights for addressing the ongoing scientific discourse. This study therefore focuses on these two types of graphitic tectonites. Based on detailed field investigations, we integrate petrographic observations, electron backscatter diffraction (EBSD), X-ray diffraction (XRD), high-resolution transmission electron microscopy (HRTEM), micro-laser Raman spectroscopy, carbon isotope analysis, and total organic carbon (TOC) analysis to comprehensively examine the occurrences, deformation mechanisms, nanostructural characteristics, and carbon sources of graphite in the shear zone. Moreover, we quantitatively characterize the structural ordering of graphite at the nanoscale and explore the graphitization process in shear zones, with implications for crustal weakening and the genesis of inorganic carbon.

## 2. Geological Background and Deformation Characteristics of Shear Zones

**Figure 1:**
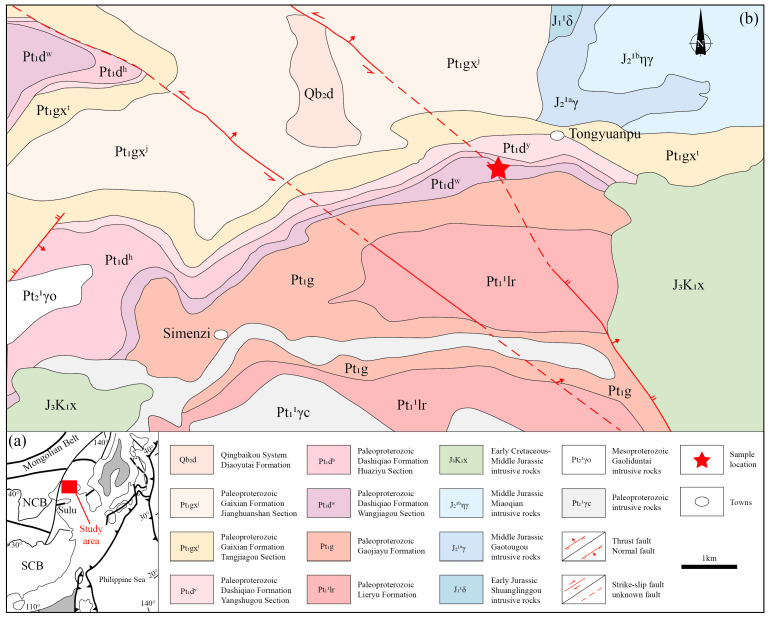
(**a**) Geological map of Liaodong Peninsula. (**b**) Geological map of Tongyuanbao Area. NCB—North China Block. SCB—South China Block.

The Liaodong region is situated on the northeastern margin of the North China Craton, within the northern segment of the Jiao-Liao-Ji orogenic belt (Figure 1a). It is primarily composed of a suite of granitic rocks (Liaoji Granite) and a thick metamorphosed sedimentary sequence known as the Liaohe Group. The Liaohe Group, from bottom to top, is divided into the Langzishan, Lieryu, Gaojiayu, Dashiqiao, and Gaixian formations. This sequence collectively consists of biotite-monzonitic leptynite, biotite-plagioclase leptynite, dolomitic marble, diopsidite, and carbonaceous slate [27].The Liaodong region has undergone complex tectonic evolution since the Archean, recording numerous events from the Paleoproterozoic to the Meso-Cenozoic. For example, the Liaohe Group, widely distributed in the northern part of the region, is regarded as a typical area of Paleoproterozoic plate collision and amalgamation, forming the Paleoproterozoic orogenic belt in the northern Liaodong Peninsula [28,29,30,31]. Since the Mesozoic, the Liaodong region has experienced intense tectonic reworking, recording the formation of small to medium-sized basins and extensive volcanic-magmatic activities. During the Early to Middle Jurassic, this region underwent fold-thrust compression [32,33,34]. Extensive Early Cretaceous extensional tectonics make it a representative area for the destruction of the North China Craton [35,36,37].

**Figure 2:**
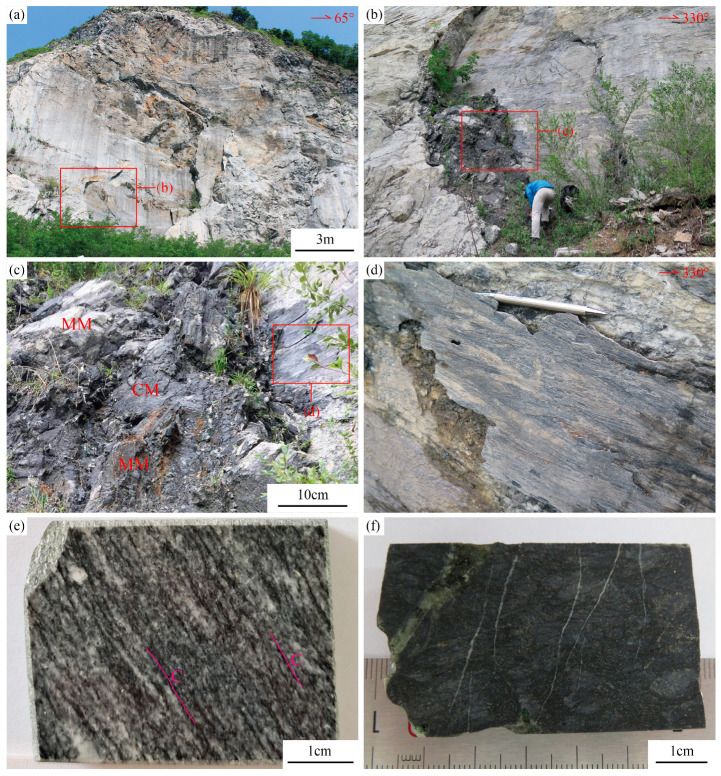
Deformation characteristics of Tongyuanpu shear zone. (**a**–**d**) Outcrop characteristics in the shear zone. (**e**) Photo of MM hand specimens with C-foliation. (**f**) Photo of CM hand specimens. MM—mylonitic marble. CM—cataclastic marble.

The graphitic shear zone investigated in this study is located in the Tongyuanpu area at the eastern end of Liaodong Peninsula, hosted within the marble of Wangjiagou Section of Paleoproterozoic Dashiqiao Formation (Figure 1b). Field investigations reveal that this shear zone generally strikes nearly north–south, dips steeply to the west with a dip angle greater than 70°, and exhibits relatively planar shear surfaces. Slickenlines and steps composed of quartz and graphite are developed on these surfaces, indicating sinistral strike-slip shearing with a reverse component (Figure 2a–d). Based on tectonic stress field calculations, reference [33] determined that the maximum principal compressive stress field of this shear zone is oriented NW–SE. Combined with regional magmatic chronology, these findings suggest the shear zone formed during the transition from compressional to extensional tectonics in the Late Jurassic to Early Cretaceous and is closely related to the destruction of the North China Craton.

Further observations reveal that this shear zone contains two sets of graphitic tectonites with varying degrees of deformation (Figure 2c,e,f). The intensely sheared mylonitized marble (MM) is distributed along the shear zone. This rock exhibits alternating black and white bands, with pervasive development of C-foliation. This foliation is composed of elongated and aligned mineral aggregates including calcite, quartz, and graphite. Some calcite forms σ-type rotated porphyroclasts, indicating significant sinistral shearing (Figure 2e). Additionally, an approximately 0.2-meter-wide zone of cataclastic marble (CM) is exposed in the core of the shear zone, coexisting with and mutually wrapping the MM (Figure 2c,f). This rock appears predominantly black and has undergone intense brittle fracturing, with the matrix content exceeding 50%. This matrix is mainly composed of finely granulated calcite, with graphite and pyrite distributed along grain boundaries. This rock is also crosscut by later multi-phase calcite veins (Figure 2f). These characteristics imply that the CM may have developed from pre-deformed MM in the same shear zone.

A detailed analysis of micro- to ultra-microstructural deformation characteristics and nanostructural features of graphite was conducted on the two types of graphitic tectonites from the study area. For EBSD fabric analysis of the MM samples, oriented thin sections were prepared by cutting and polishing perpendicular to the foliation and parallel to the lineation (*XZ* plane).

## 3. Experimental Methods

### 3.1. Electron Backscatter Diffraction

The electron backscatter diffraction (EBSD) analysis was performed on a FEI Quanta 450 field emission gun scanning electron microscope coupled with an Oxford Nordlys F+ EBSD detector at Rock Structure Analysis Laboratory, Key Laboratory of Deep Earth Dynamics, Institute of Geology, Chinese Academy of Geological Sciences. *XZ* sections for EBSD mapping were chemically and mechanically polished with a 0.05 µm alumina suspension for about two hours. The analysis was performed at an accelerating voltage of 20 kV, and a working distance of 16 mm. Samples were tilted at 70°, and a step size of 6 µm was used for EBSD mapping. All collected data were processed using the HKL Channel 5 software package, and all pole figures were presented as equal area, lower hemisphere projections.

### 3.2. Micro-Laser Raman Spectroscopy

The micro-laser Raman spectroscopy analysis was conducted at the Beijing Key Laboratory of Mineral Environmental Function, Peking University, using a Renishaw inVia Reflex instrument (Gloucestershire, UK) with a laser wavelength of 532 nm and a scanning range of 0–2000 cm^−1^. The acquired Raman spectra were processed by peak deconvolution and fitting. Parameters of the characteristic peaks, including peak position, peak area, full width at half maximum (FWHM), and peak intensity (height), were obtained [38]. Based on these parameters, the metamorphic peak temperature (T_MP_) of samples was calculated using the formula proposed by reference [39]: T_MP_(°C) = 737.3 + 320.9R_1_ − 1067R_2_ − 80.638R_1_^2^, where R_1_ = D_1_/G_intensity_ and R_2_ = D_1_/(G + D_1_ + D_2_)_area_.

### 3.3. X-Ray Diffraction

The X-ray diffraction (XRD) analysis was conducted at the Analysis and Testing Center of Henan Polytechnic University, using a SmartLab rotating anode X-ray diffractometer. The tube voltage was set at 90 kV (Max), and the X-ray tube current was 55 mA. A step-scan mode was employed with a step size of 0.2°, a scanning speed of 5°/min, and a scanning range of 5–70°. The (002) peak near 2θ = 26° was fitted using a multi-peak fitting approach with a Gaussian function to obtain its position, area, full width at half maximum (FWHM), and intensity. The Bragg equation was then utilized to calculate L_c_, d_002_, and β_002_. Finally, from which the degree of graphitization was determined [40].

### 3.4. High-Resolution Transmission Electron Microscopy

High-resolution transmission electron microscopy (HRTEM) imaging was performed at the Electron Microscopy Laboratory of Peking University using a Tecnai T20 transmission electron microscope operated at an accelerating voltage of 200 kV, with point and line resolutions of 0.24 nm and 0.102 nm, respectively. For the acquired HRTEM images, scale calibration was first carried out by drawing a straight line along the scale bar direction. After selecting the target analysis area, fast Fourier transform (FFT) was applied to the original image to convert spatial domain data into the frequency domain, producing a frequency spectrum image. Based on noise frequency characteristics, bandpass (or low-pass) filtering was used to process the frequency domain image, eliminating high-frequency noise interference. The inverse Fourier transform (IFFT) was then applied to convert the filtered frequency domain data back to the spatial domain, resulting in a denoised lattice fringe image. Finally, straight lines were drawn perpendicular to the lattice fringes, and the lattice spacing in the target area was calculated as the ratio of the total spacing length to the number of intervals. To ensure accuracy, statistical analysis was performed using multiple area measurements (≥35), with strict control of the signal-to-noise ratio and focus calibration to minimize measurement errors.

### 3.5. Total Organic Carbon

The total organic carbon (TOC) analysis was conducted at Compass Testing Company using a German Elementar vario TOC select instrument. The approximate TOC concentration range of the samples was 0–30%. Each sample was tested twice, and the average value was taken.

### 3.6. Carbon Isotope

Carbon isotope testing was completed at the State Key Laboratory of Ore Deposit Geochemistry, Institute of Geochemistry, Chinese Academy of Sciences. The analysis was performed using a MAT 253 stable isotope ratio mass spectrometer manufactured by Thermo Fisher Scientific (Waltham, MA, USA). Prior to testing, the collected rock samples were uniformly ground to 200 mesh to ensure sample homogeneity. For organic carbon isotope testing of graphite, acid washing (15% HCl, 48 h) was applied before analysis to remove inorganic carbon. During testing, a small amount of sample (a few milligrams) was combusted with copper oxide in sealed quartz tubes at 1000 °C for 6 h. The resulting CO_2_ was purified through condensation and analyzed using a MAT 253 gas source isotope ratio mass spectrometer (Thermo Fisher Scientific, Dreieich, Germany). For inorganic carbon isotope testing of calcite, a GasBench II device (Thermo Fisher Scientific, Dreieich, Germany) was used. Samples were purged with helium flow for 8 min, followed by the addition of phosphoric acid to generate CO_2_ for analysis. The samples reacted with the acid at 72 °C for at least 4 h, after which the δ^13^C values were measured using the MAT 253 mass spectrometer. All carbon isotope results are reported relative to the VPDB standard.

## 4. Results

### 4.1. Microscopic Deformation and Lattice Preferred Orientation Characteristics of Graphitic Tectonites

#### 4.1.1. Microscopic Deformation Characteristics

**Figure 3:**
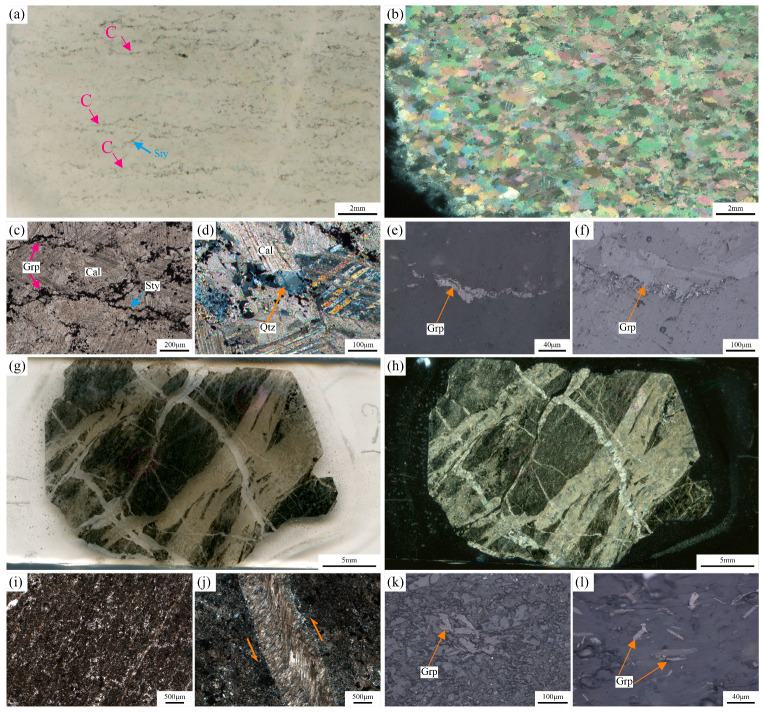
Microscopic petrographic characteristics of graphitic tectonites. (**a**,**c**,**g**,**i**) Transmitted single polarized light. (**b**,**d**,**h**,**j**) Transmitted orthogonal polarization; (**e**,**f**,**k**,**l**) Reflects single polarized light. C—C-foliation. Cal—calcite. Grp—graphite. Qtz—quartz. Sty—stylolite.

The MM samples are primarily composed of calcite and graphite, with minor amounts of quartz. The calcite grains have undergone recrystallization, exhibiting irregular grain boundaries. The long axes of these grains are approximately parallel to the C-foliation or intersect it at small angles. These calcites show well-developed twins and are relatively coarse (>0.5 mm) (Figure 3a–c). Quartz is mainly distributed along calcite grain boundaries, with smaller grain sizes (~0.1 mm), and exhibits wavy extinction under cross-polarized light (Figure 3d). Most graphite occurs as euhedral to subhedral flaky aggregates, with grain long axes ranging from 10 to 50 μm (Figure 3e). These aggregates are often oriented along calcite grain boundaries (Figure 3a,c), and display two sets of different extension directions. One set is parallel to the long axis of calcite, forming continuous or discontinuous slip surfaces. Collectively, these graphite aggregates define the C-foliation, indicating that graphite formed synchronously during the mylonitization process (Figure 3a). The other set locally intersects the slip surfaces at small angles, and graphite aggregates show a serrated distribution, which is associated with pressure-solution stylolites (Figure 3c,f). These stylolites were cut by C-foliation-parallel graphite aggregates, suggesting two separate formation events, with stylolite development preceding the main graphite crystallization.

In CM, mineral grains are intensely comminuted and crosscut by multiple generations of calcite veins, making mineral identification challenging. Grain sizes are generally < 30 μm (Figure 3g–i). Sheared calcite veins are observed with fibers on both sides perpendicular to the vein walls; their central portions show bending deformation, indicating sinistral shear consistent with the outcrop-scale shear sense (Figure 3j). Graphite occurs as euhedral to subhedral flakes, distributed non-directionally within the cataclastic matrix as individual grains that do not exhibit slip characteristics. These graphite crystals exhibit better-developed morphologies than those in MM, with long axes ranging from 40 to 100 μm (Figure 3k,l).

#### 4.1.2. EBSD Lattice Preferred Orientation Characteristics

**Figure 4:**
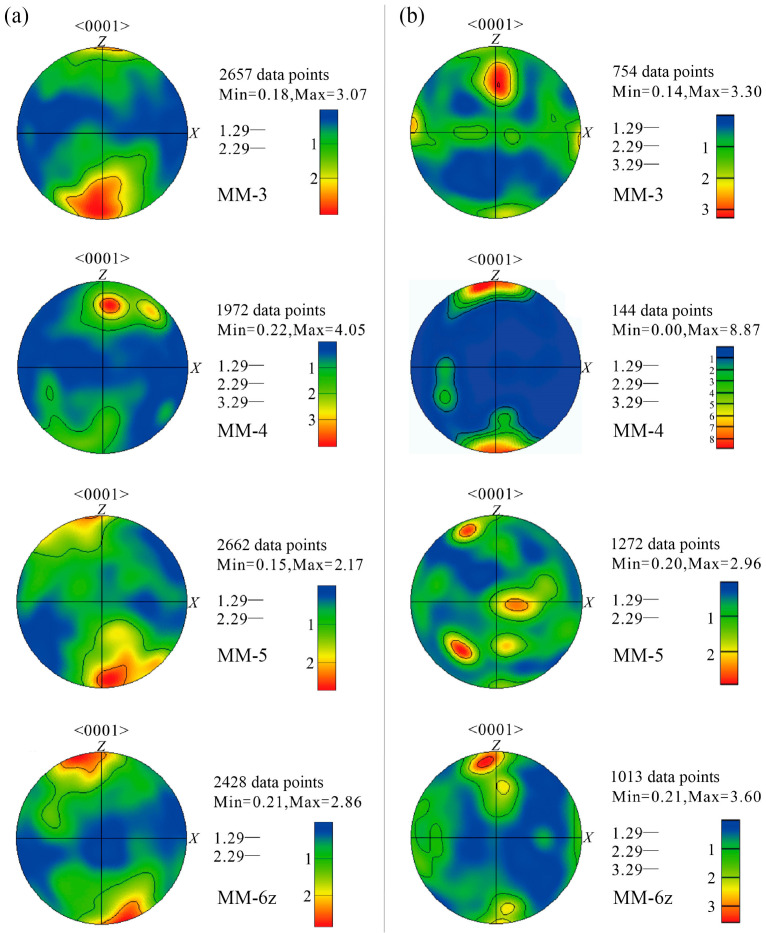
Calcite (**a**) and graphite (**b**) *c*-axis fabrics in MM. The measured points are single-grain points (including old grains and new recrystallized grains).

Due to intense brittle deformation and the resulting poor mineral orientation in the CM samples, this study primarily selected four sets of MM samples (MM-3, MM-4, MM-5, MM-6z) for analysis of the lattice preferred orientation (LPO) of calcite and graphite grains using electron backscatter diffraction (EBSD) (Figure 4). For calcite, fine-grained minerals were selected as the analysis targets during the experiment, with the projected element being the *c*-axis (0001) plane. The results show that calcite generally forms point maxima near the *Z*-axis (Figure 4a). Specifically, the maxima of MM-3 are distributed across all four quadrants, with a primary concentration in the third and fourth quadrants; MM-4 exhibits maxima mainly in the first quadrant; MM-5 and MM-6 show maxima predominantly in the second and fourth quadrants. Overall, the well-developed LPO in MM samples reflects basal <a> slip, suggesting a deformation temperature below 200 °C [41].

The LPO analysis of graphite reveals point maxima near the *Z*-axis (Figure 4b). Specifically, the maxima of MM-3 are mainly distributed in the first and second quadrants; MM-4 and MM-5 exhibit maxima across all four quadrants; MM-6z shows maxima predominantly in the second quadrant. These results are largely consistent with the EBSD findings for calcite in the MM samples (Figure 4a), indicating that calcite and graphite share highly similar slip system types, both exhibiting basal <a> slip. Meanwhile, it can also be seen that the point maxima of some samples deviated from the *Z*-axis (e.g., sample MM-5), which might be the result of the superposition of graphite fabrics formed in two different events.

### 4.2. TOC Contents and Characteristics of Carbon Nanostructures

#### 4.2.1. TOC Contents

TOC analysis was conducted on the two types of tectonites from the study area, with the results presented in Table 1. The TOC values of the MM samples are relatively low, ranging from 8.04 wt‰ to 8.35 wt‰ (average 8.20 wt‰). In contrast, the CM samples exhibit higher TOC values, ranging from 23.59 wt‰ to 23.74 wt‰ (average 23.67 wt‰).

#### 4.2.2. XRD Spectral Characteristics

**Figure 5:**
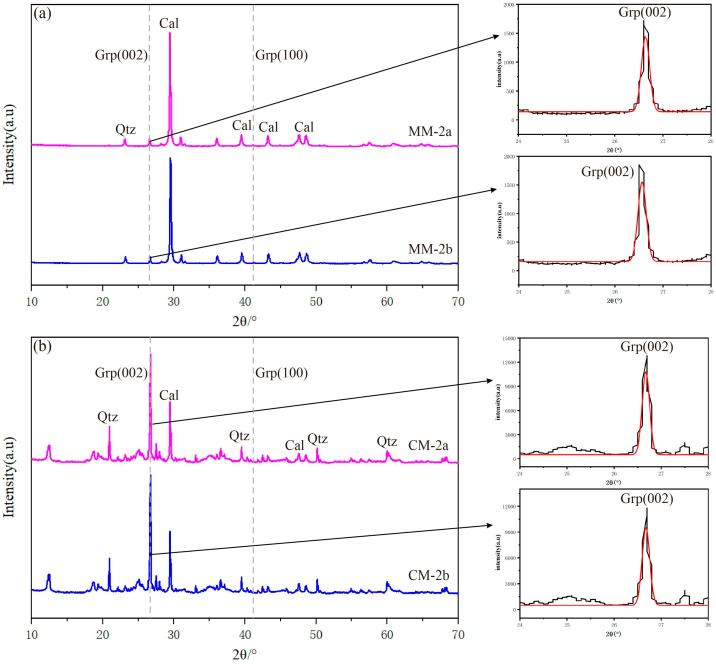
XRD pattern and graphite peak fitting diagrams for MM (**a**) and CM (**b**). Cal—calcite (card number 99–0022). Grp—graphite (card number 99–0057). Qtz—quartz (card number 99–0088). Purple, blue and black lines—experimental curves. Red line—fitting curve.

The XRD patterns indicate that the mineral components in the two types of tectonites are calcite, quartz and graphite (Figure 5). Peaks observed at 2θ = 26.5° and 2θ = 42.3°are associated with graphite crystals, corresponding to the (002) and (100) crystallographic planes of graphite, respectively. Further observations reveal that the peak at 26.5° is the sharpest and most prominent, reflecting a high degree of ordering and excellent crystallinity in the interlayer stacking of graphite along the c-axis. In contrast, the peak at 42.3° is relatively weak, indicating either weaker orientation consistency in the in-plane atomic arrangement of graphite crystals (along the a-axis) or refinement of the crystallites within the basal plane. Furthermore, the graphite diffraction peaks in CM display sharper shapes and higher intensities compared to those in MM, suggesting a relatively higher degree of graphitization.

In the study of graphite nanostructural characterization, crystallite thickness (L_c_), interlayer spacing (d_002_), and degree of graphitization (DG) are widely regarded as key parameters for evaluating structural ordering. The relevant parameters were calculated using formulas from reference [42] (Table 2). The results show that in MM, L_c_ ranges from 391 to 403 × 10^−10^ m, d_002_ ranges from 3.344 to 3.353 × 10^−10^ m, and DG ranges from 85% to 90%. In CM, L_c_ ranges from 425 to 432 × 10^−10^ m, d_002_ ranges from 3.338 to 3.339 × 10^−10^ m, and DG ranges from 97% to 99%. Overall, both types of samples exhibit a high degree of graphitization, while the graphite in CM shows a higher degree of crystallization compared to that in MM.

#### 4.2.3. Raman Spectral Characteristics and Peak Metamorphic Temperature

**Figure 6:**
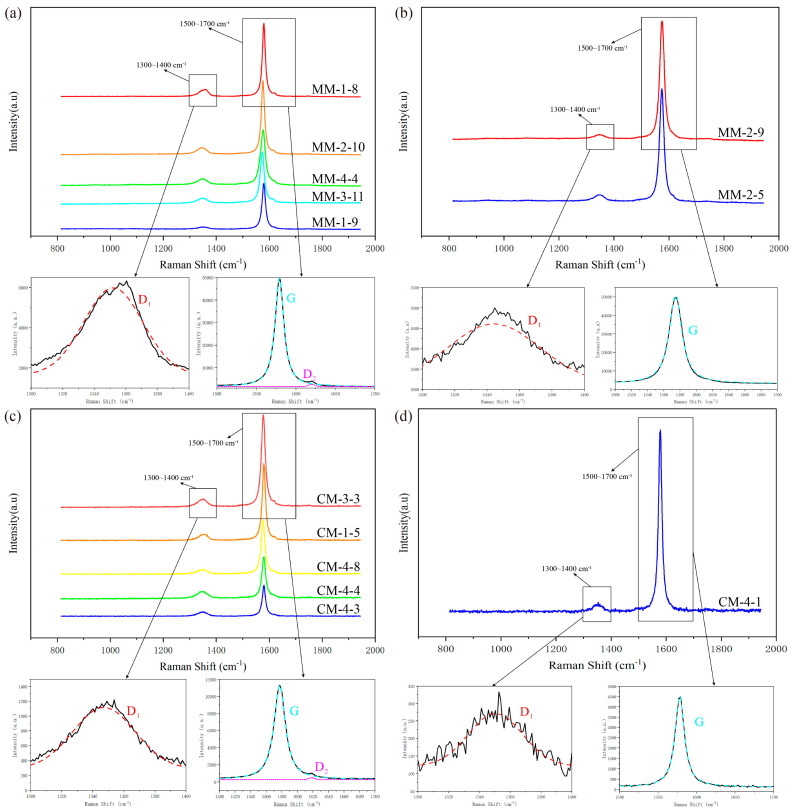
Representative examples of Raman spectra of graphite for MM (**a**,**b**) and CM (**c**,**d**).

The Raman analysis results show that a sharp G peak is observed near 1580 cm^−1^ in all samples, indicating well-ordered graphite crystals and serving as a characteristic marker of crystallinity. A distinct D_1_ peak appears near 1350 cm^−1^, with a relatively broad and gentle shape, reflects structural disorder, including two-dimensional plane defects and potential heteroatoms, suggesting the presence of lattice imperfections. Some samples show a weak D_2_ peak near 1620 cm^−1^, characterized by low intensity and narrow width, partially overlapping with the G peak. This feature may be associated with disorder in the graphite lattice hexagonal rings and structural defects [38,43] (Figure 6). Collectively, these spectral characteristics confirm that the graphite in the marble shear zone exhibits typical crystalline features alongside a certain degree of lattice defects.

The metamorphic peak temperature (T_MP_) of the two graphitic tectonits were calculated using the graphite Raman geothermometer (Table 3). To minimize errors and adequately quantify the degree of graphitization, more than five graphite grains from each sample were selected for testing. The results indicate that the T_MP_ of MM range from 588 °C to 673 °C (average 626 °C), while those from CM range from 540 °C to 682 °C (average 608 °C). Overall, both types of tectonites have undergone relatively high-grade metamorphism.

**Figure 7:**
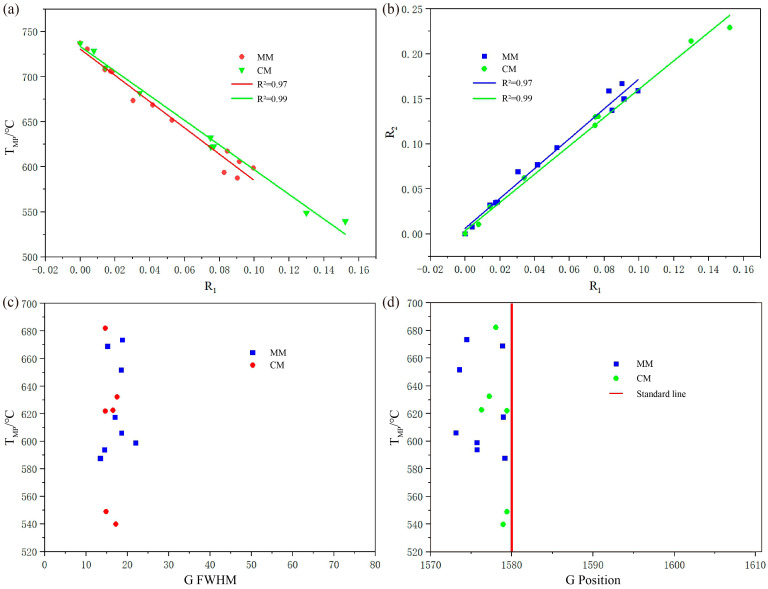
Diagram of the Raman parameter relationship of graphite in two types of tectonites. (**a**) T_MP_ vs. R_1_ ratio. (**b**) R_2_ ratio vs. R_1_ ratio. (**c**) T_MP_ vs. G FWHM. (**d**) T_MP_ vs. G position.

A series of relevant parameters were also calculated and plotted (Table 3; Figure 7). In MM samples (MM18–MM44), R_1_ values range from 0.03 to 0.10 (average 0.07), and R_2_ values range from 0.07 to 0.17 (average 0.13). In CM samples (CM15–CM48), R_1_ values range from 0.03 to 0.15 (average 0.08), and R_2_ values range from 0.06 to 0.23 (average 0.15). The plotted results indicate a significant negative correlation between T_MP_ and R_1_ (with correlation coefficients R^2^ of 0.97 and 0.99, respectively) (Figure 7a), and a significant positive correlation between R_2_ and R_1_ (with correlation coefficients R^2^ of 0.98 and 0.99, respectively) (Figure 7b). These data show an inverse correlation between the degree of graphitization and structural disorder, thus reflecting a corresponding increase in metamorphic temperature.

FWHM of the G peak in MM samples mostly ranges from 13 to 23, whereas in CM samples, it predominantly falls between 14 and 18. The narrower FWHM in CM reflects a more ordered graphite structure (Figure 7c). Moreover, the G peak positions in both tectonites are downshifted relative to the standard 1580 cm^−1^ line, with the CM values being closer to the standard than those of MM (Figure 7d). Overall, these results indicate that graphite in CM exhibits higher structural ordering and crystallinity than that in MM.

#### 4.2.4. HRTEM Characteristics

**Figure 8:**
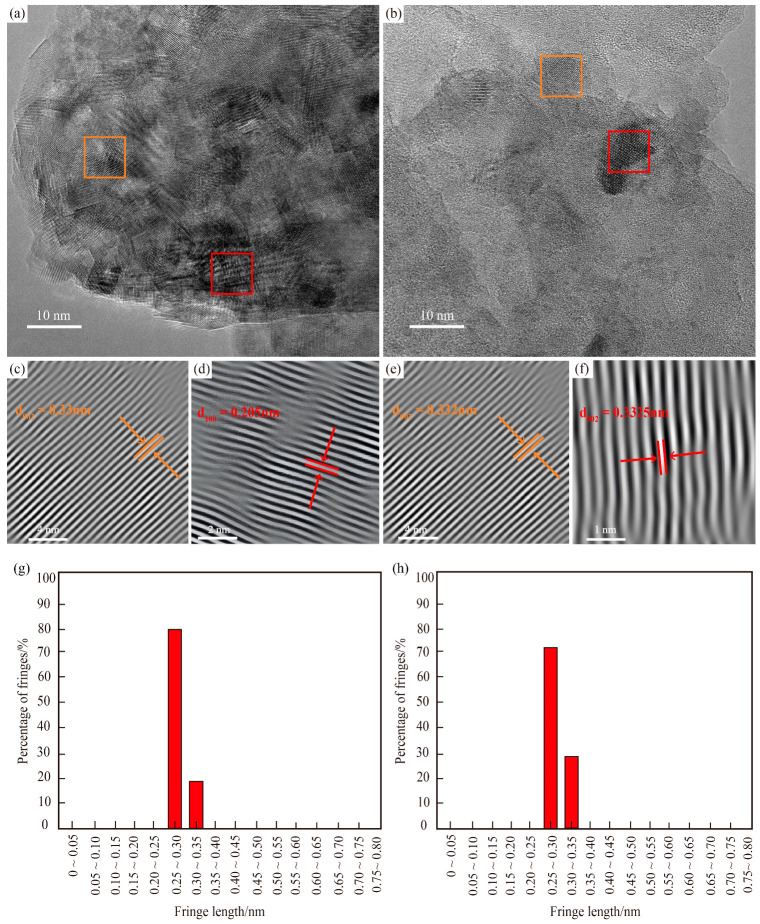
HRTEM characteristics of two types of tectonites. (**a**,**c**,**d**) HRTEM micrograph for MM. (**b**,**e**,**f**) HRTEM micrograph for CM. (**g**) Fringe length distribution diagram for MM. (**h**) Fringe length distribution diagram for CM.

HRTEM images reveal that graphite in both MM (Figure 8a) and CM (Figure 8b) primarily occurs as thin, stacked flakes. Under electron beam-induced crystallization, larger flake areas gradually transform into smaller crystals. A small number of dark, rounded crystals are observed in stacked arrangements, and their crystal morphology becomes more defined under electron beam irradiation, indicating good crystallinity and uniform phase distribution. Both samples contain upright carbon layers with widely distributed, straight (002) lattice fringes at the nanoscale (Figure 8c,e). The measured interlayer spacing (d_002_) is 0.33 nm for MM, and 0.332 nm for CM, confirming that both have essentially attained the standard graphite structure (d_002_ = 0.336 nm). A limited number of curved fringes are also observed (Figure 8d,f), clearly showing carbon layer stacking and misalignment. This suggests that while carbon atoms within individual layers have formed a graphite structure, interlayer rotations exist at certain angles [47]. A total of 45 random points were measured for MM and 35 for CM, with the statistical distributions of fringe lengths plotted accordingly (Figure 8g,h). The histograms show that fringe lengths in both samples predominantly range from 0.25 to 0.35 nm, with the majority (80% for MM and 71% for CM) concentrated between 0.25 and 0.30 nm. The portions closest to the standard graphitic spacing (0.336 nm) account for 20% and 29%, respectively. These relatively high proportions further confirm that the carbonaceous material has largely evolved toward a well-ordered graphite structure.

### 4.3. Carbon Isotopic Composition

Carbon stable isotopes provide a unique and powerful tool for deciphering the sources of carbon in different geological environments. This study conducted a systematic analysis of the carbon isotope composition of two types of tectonites from the marble shear zone, including the organic carbon isotope composition of graphite (δ^13^C_ORG_) and the inorganic carbon composition of calcite (δ^13^C_CAL_). By analyzing the characteristics of the δ^13^C values, we aim to reveal the carbon sources of graphite in this shear zone. The test results show that the δ^13^C values of the two samples are relatively close (Table 4). δ^13^C_ORG_ in MM ranges from −8.06‰ to −7.31‰, with an average of −7.69‰. In CM, δ^13^C_ORG_ ranges from –12.47‰ to −10.47‰, with an average of −11.47‰. These data indicate that the graphite in both tectonites has a relatively heavy carbon isotope composition, which differs significantly from the δ^13^C composition of regional metamorphic graphite (−25.9‰ to −13.69‰, data from references [48,49,50]). Instead, it aligns with the δ^13^C characteristics of fluid-derived graphite (> −16‰) [11,16,51]. Integrating the δ^13^C_CAL_ composition characteristics of graphite, the highly graphitized features revealed by carbon nanostructural characterization and the high metamorphic peak temperatures recorded by the graphite geothermometer, this study concludes that the formation of graphite in both samples is related to fluid activity rather than an organic origin. Furthermore, the relatively lighter carbon isotope composition of δ^13^C_ORG_ in CM compared to MM may be associated with multi-phase fluid activity during the cataclasis process [52] (Figure 3g,h). Additionally, the δ^13^C_CAL_ in MM ranges from −2.65‰ to −2.61‰, with an average of −2.63‰, while in CM it ranges from –3.87‰ to −3.86‰, with an average of −3.865‰. Both are generally consistent with the carbon isotope composition of marine carbonate rocks [53], suggesting a unified source for the calcite in both tectonites.

## 5. Discussion and Conclusions

### 5.1. The Influence of Deformation–Metamorphism on Graphitization

Shear zones are typically regional structural weak zones with relatively concentrated fluid activity. Rocks within shear zones undergo intense deformation under shear stress, often accompanied by metamorphism, and the degree of metamorphism generally increases with the intensity of deformation [54]. Therefore, precisely constraining the deformation-metamorphism relationship is crucial for revealing the deformation processes of shear zones and the genesis of graphite. Our microstructural and EBSD analyses reveal that both calcite and graphite in MM exhibit similar deformation behaviors by microstructural investigations and EBSD analysis. These minerals undergo elongation and directional arrangement, forming the C-foliation, indicating that they experienced shear deformation during the same period (Figure 2 and Figure 3). Moreover, their maxima distribution near the *Z*-axis indicates that the deformation temperature was below 200 °C (Figure 4). In contrast, the Raman geothermometer results reveal that the metamorphic peak temperature of graphite in these samples can reach up to 626 °C (Table 3). This discrepancy highlights an inconsistency between deformation temperature and metamorphic temperature in the studied shear zone, significantly deviating from the previously proposed co-evolution trend [10,54].

Based on previous research, we propose that the discrepancy may be related to the Raman geothermometer employed. The thermometers proposed by references [39,55] are empirical formulas that primarily consider the influence of metamorphism (heat) on the crystallinity of carbonaceous materials and Raman structural parameters [56]. However, during the activities in shear zones, shear stress can not only cause physical changes (deformation) in rocks, but also induce chemical changes in rock minerals by lowering the activation energy barriers for reactions, enabling them to proceed at lower temperatures [20]. Previous studies have found that high-rank anthracites require heating to at least 2200 °C under normal pressure to exhibit graphitization features, whereas anthracites found in some regions can undergo graphitization at temperatures as low as 300–500 °C, significantly lower than the temperatures observed in experimental settings [57,58]. Subsequent high-temperature and high-pressure deformation experiments conducted by references [59,60,61] revealed that graphitization in coal occurs specifically under shear deformation, not coaxial stress.

Therefore, during deformation-metamorphism processes, tectonic stress—particularly shear stress—may reduce the activation energy barrier required for carbon atom diffusion and structural rearrangement by accumulating local strain energy. This, in turn, lowers the temperature required for graphitization, accelerates the graphitization process of carbonaceous materials, and facilitates the formation of well-crystallized graphite at lower temperatures. Molecular dynamics simulations also demonstrate that amorphous carbon undergoes a phase transition from a disordered state to a layered ordered structure under shear stress [62,63]. In addition, Barzoi [64] conducted a series of experiments to investigate the impact of shear stress on the accuracy of graphite geothermometers. The results showed that the temperature error calculated using graphite geothermometers for strained and unstrained zones could be as high as 150 °C. Additionally, significant discrepancies were observed between temperatures calculated based on graphite crystallinity and those derived from the chemical composition of chlorite and chloritoid. Therefore, for graphite or carbonaceous materials in shear zones that have undergone intense shear deformation, directly applying Raman parameters to calculate peak metamorphic temperatures without considering the influence of tectonic stress may lead to inconsistencies between metamorphic and deformation temperatures.

Additionally, the presence of quartz and, more importantly, graphite, may affect the activity of slip systems “extending” basal <a> to higher temperatures. Krabbendametal proposed that the presence of graphite can inhibit grain boundary migration of other minerals (e.g., quartz), stabilize grain size, and enhance grain boundary sliding through dislocation creep [65]. Therefore, once the crystalline graphite formed, it became the primary site of strain concentration within the shear zone. This allowed rocks in the shear zone to undergo significant overall plastic strain, while individual minerals such as calcite experienced relatively limited deformation, primarily manifesting as basal <a> slip. This strain-weakening behavior will also lead to a discrepancy between the deformation temperature and the metamorphic temperature.

**Figure 9:**
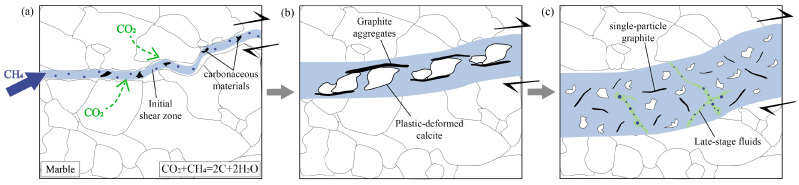
Sketch diagram of the graphite enrichment process in the shear zone. (**a**) Carbonaceous materials formed by high-temperature fluids. (**b**) Graphite aggregates arranged in a directional manner during ductile deformation. (**c**) Dispersed single-particle graphite in brittle deformation.

Based on carbon isotope characteristics, this study proposes that the formation of graphite in Tongyuanpu shear zone involved multiple stages of metamorphic fluid activity and shear deformation, respectively. During the Late Mesozoic, the Liaodong Peninsula experienced intense cratonic destruction [36], with the tectonic regime transitioning from compression to extension [33]. This process led to the development of NW–SE trending shear zones in the Wangjiagou Formation marble of the Tongyuanpu area. In the early stages of shear zone development, intense regional mantle-derived fluid activity [35,37] allowed high-temperature CH_4_-rich fluids from deep sources to migrate along the shear zone. These fluids encountered CO_2_ generated by pressure dissolution in the surrounding rocks, triggering reduction reactions that formed carbonaceous materials [9,11] (Figure 9a) with relatively heavy carbon isotope compositions [52].With the continuous action of shear stress, the structure of these carbonaceous materials gradually transformed from a disordered state to a layered ordered arrangement, resulting in graphitization and following strain concentration. Consequently, the long axes of early stylolite graphite progressively rotated and aligned parallel to the shear zone boundaries, forming localized micro-shear slip bonds (Figure 9b). And the overall crystal-plastic deformation is characterized by basal <a> slip under low-temperature conditions, which reflects strain weakening facilitated by the presence of graphite (Figure 4).

### 5.2. The Influence of Deformation Mechanisms on Graphitization

The coexistence of mylonites and cataclasites indicates that Tongyuanpu shear zone has undergone deformation at different structural levels during its development. According to carbon isotope data, the δ^13^C_ORG_ values of both MM and CM are greater than −16‰, indicating a consistent fluid-derived origin for the graphite in both tectonites. Additionally, the δ^13^C_CAL_ values are all around −3‰, further supporting a unified source for inorganic carbon. Furthermore, both tectonites initially experienced high-temperature metamorphic fluid activity, with average metamorphic peak temperatures of 626 °C and 608 °C, respectively. These characteristics suggest that both tectonites originated from the same protolith, and cataclasites developed from pre-deformed mylonites in the same shear zone. However, the graphite carbon content in CM is significantly higher than that in MM, and it exhibits a relatively higher degree of graphitization and structural ordering. This study attributes these differences to the deformation mechanisms of the two tectonites at different structural levels.

At deeper ductile deformation level, shear zones are dominated by crystal-plastic deformation. Shear stress promotes the oriented alignment of calcite and graphite, leading to the formation of MM (Figure 9b). However, the presence of graphite causes strain concentration and significant rheological weakening, resulting in calcite crystal deformation primarily through low-temperature basal <a> slip (Figure 4). As tectonic uplift and intense fluid activity progressed, the deformation mechanism of this shear zone gradually transitioned to brittle friction. Intense frictional sliding further facilitated the transformation of carbonaceous materials into crystalline graphite [5,18]. Consequently, both the content of organic carbon and the degree of graphitization increased continuously. Rotational shear friction experiments on carbon-bearing marble further confirm that brittle deformation and multi-stage deformation more readily enhance the degree of graphitization in marble [26].

Based on comprehensive research, the formation of graphite in the Tongyuanpu shear zone is controlled by multiple geological factors. First, reduction reactions involving high-temperature metamorphic fluids provided the essential carbon source for graphite formation. Second, ductile shear deformation (mylonitization) accelerated the graphitization of carbonaceous materials, and the aligned graphite aggregates enabled the shear zone to accommodate significant strain through grain boundary sliding. Third, frictional sliding during brittle deformation (cataclasis) further promoted the transformation of carbonaceous materials into crystalline graphite. Throughout these processes, tectonic stress likely reduced the temperature required for graphitization by accumulating local strain energy, providing new insights into the low-temperature genesis of graphite in shear zones. This study also offers important implications for regional graphite mineral exploration.

## Figures and Tables

**Table 1:** TOC contents in two types of tectonites.

Samples	TOC Content/wt‰
MM-1	8.35
MM-2	8.04
CM-1	23.74
CM-2	23.59

**Table 2:** Graphite nanostructural parameters for XRD data.

Samples	K	λ	2θ/°	β_002_/°	d_002_ /10^−10^ m	L_c_ /10^−10^ m	DG/%
MM-2a	0.89	1.5406	26.64	0.20	3.344	403	90
MM-2b	0.89	1.5406	26.57	0.21	3.353	391	85
CM-2a	0.89	1.5406	26.67	0.19	3.339	432	99
CM-2b	0.89	1.5406	26.68	0.19	3.338	425	97

**Table 3:** Raman spectral parameters of graphite and peak metamorphic temperatures.

Samples	Peak Type	Peak Area	FWHM/cm^−1^	Peak Intensity/cm^−1^	Peak Position/cm^−1^	R_1_	R_2_	T_MP_/°C	Vibration Type
MM18	D_1_	203,788.83	44.14	4337.02	1351.96	0.09	0.17	588	G –in-plane E_2g_ bond-stretching motion of sp^2^ carbons [44,45]. D_1_ and D_2_ –A _1g_ symmetrical vibration motion of sp^2^ carbons [44,45,46].
	G	1,007,350.99	13.47	48,012.90	1579.14				
	D_2_	10,166.19	9.37	1018.88	1619.70				
MM19	D_1_	59,388.36	44.48	1254.21	1348.92	0.04	0.08	669	
	G	713,173.12	15.23	30,115.98	1578.85				
	D_2_	2211.27	5.94	349.80	1620.52				
MM25	D_1_	136,136.60	54.22	2358.65	1344.64	0.05	0.10	652	
	G	1,283,158.31	18.53	44,609.54	1573.58				
	D_2_	—	—	—	—				
MM29	D_1_	103,689.01	66.79	1458.36	1344.12	0.03	0.07	673	
	G	1,398,986.07	18.83	47,868.37	1574.47				
	D_2_	—	—	—	—				
MM210	D_1_	206,820.96	49.16	3952.65	1344.40	0.08	0.16	594	
	G	1,080,394.53	14.52	47,803.75	1575.74				
	D_2_	14,482.68	13.41	1014.62	1615.55				
MM311	D_1_	173,448.31	53.47	3047.67	1346.90	0.09	0.15	606	
	G	962,372.91	18.59	33,349.44	1573.12				
	D_2_	20,560.11	16.88	1144.17	1614.12				
MM44	D_1_	232,729.25	61.45	3558.03	1346.27	0.10	0.16	599	
	G	1,222,144.28	22.06	35,775.14	1575.72				
	D_2_	8841.40	13.41	619.32	1616.42				
CM15	D_1_	31,510.07	43.04	687.83	1350.43	0.07	0.13	622	
	G	209351.83	14.67	9172.57	1579.40				
	D_2_	1267.16	7.45	159.74	1619.87				
CM33	D_1_	41,628.39	47.11	830.18	1347.14	0.07	0.12	632	
	G	301,693.34	17.51	11,094.52	1577.26				
	D_2_	2085.99	8.55	229.30	1618.38				
CM41	D_1_	6600.58	41.34	149.99	1350.38	0.03	0.06	682	
	G	100,000.76	14.63	4395.12	1578.05				
	D_2_	—	—	—	—				
CM43	D_1_	23,518.86	46.47	475.42	1346.88	0.13	0.21	549	
	G	84,423.01	14.83	3659.58	1579.39				
	D_2_	1797.93	22.48	75.12	1616.02				
CM44	D_1_	40,522.82	50.66	751.39	1346.50	0.15	0.23	540	
	G	131,818.18	17.19	4936.82	1578.94				
	D_2_	4493.76	27.19	155.28	1616.72				
CM48	D_1_	25,021.11	47.59	493.89	1346.11	0.08	0.13	623	
	G	165,583.67	16.54	6443.11	1576.30				
	D_2_	1634.96	11.38	134.92	1616.91				

**Table 4:** Carbon isotopic composition in two types of tectonites.

Samples	δ^13^C_ORG_ (‰, VPDB)	δ^13^C_CAL_ (‰, VPDB)
MM-1	−7.31	−2.61
MM-2c	−8.06	−2.65
CM-1a	−12.21	−3.86
CM-1b	−10.47	−3.87

## Data Availability

The original contributions presented in this study are included in the article. Further inquiries can be directed to the corresponding author.
